# The Role of Bacterial Secretion Systems in the Virulence of Gram-Negative Airway Pathogens Associated with Cystic Fibrosis

**DOI:** 10.3389/fmicb.2016.01336

**Published:** 2016-08-30

**Authors:** Sofie Depluverez, Simon Devos, Bart Devreese

**Affiliations:** Laboratory for Protein Biochemistry and Biomolecular Engineering, Department of Biochemistry and Microbiology, Ghent UniversityGhent, Belgium

**Keywords:** infection, cystic fibrosis, pathogenesis, antimicrobial resistance, protein biosynthesis, Gram-negative bacteria

## Abstract

Cystic fibrosis (CF) is the most common lethal inherited disorder in Caucasians. It is caused by mutation of the CF transmembrane conductance regulator (CFTR) gene. A defect in the CFTR ion channel causes a dramatic change in the composition of the airway surface fluid, leading to a highly viscous mucus layer. In healthy individuals, the majority of bacteria trapped in the mucus layer are removed and destroyed by mucociliary clearance. However, in the lungs of patients with CF, the mucociliary clearance is impaired due to dehydration of the airway surface fluid. As a consequence, patients with CF are highly susceptible to chronic or intermittent pulmonary infections, often causing extensive lung inflammation and damage, accompanied by a decreased life expectancy. This mini review will focus on the different secretion mechanisms used by the major bacterial CF pathogens to release virulence factors, their role in resistance and discusses the potential for therapeutically targeting secretion systems.

## Bacterial Infections Involved in Cystic Fibrosis (CF) Lung Disease

The combination of a highly viscous, dehydrated mucus layer, defective mucociliary clearance and a number of yet unknown factors make patients with CF extremely susceptible to infections (Lipuma, 2010). *Pseudomonas aeruginosa* is the most prevalent Gram-negative species, infecting about 50% of all patients. It is detected in 25% of children, but approximately 70% of patients older than 25 years tested positive (Cystic Fibrosis Foundation, 2015). Members of the *Burkholderia cepacia* complex (Bcc) cause chronic infections in CF patients, which results in approximately 20% of the cases in fatal ‘cepacia syndrome,’ characterized by necrotizing pneumonia, bacteremia, sepsis and eventually death (Lipuma, 2010). The prevalence of Bcc is highest in adults, affecting about 4% of the patients, with *B. cenocepacia* and *B. multivorans* accounting for 70% of the Bcc infections. Several reports indicate that the incidence of *Stenotrophomonas maltophilia* in CF patients has increased considerably in recent years (Denton and Kerr, 2002). This opportunistic nosocomial pathogen is mostly recovered from adolescent patients, with a prevalence of ± 15% (Razvi et al., 2009; Cystic Fibrosis Foundation, 2015). Prevalence of *Haemophilus influenzae* is maximal at an age of 2–5 years (32%) and decreases thereafter (Cystic Fibrosis Foundation, 2015). *Achromobacter xylosoxidans* is also an emerging CF pathogen with an overall prevalence around 6% (Razvi et al., 2009).

Common to all these species is their dramatic intrinsic or acquired resistance against most of the currently employed antibiotics, making these infections extremely difficult to eradicate. Efflux pumps, biofilm formation, decreased outer membrane permeability, and inactivation of β-lactam antibiotics by chromosomally encoded β-lactamases are the main causes of resistance (Hoyle and Costerton, 1991; Waters, 2012).

## Virulence Factors

Each of the abovementioned species has its own repertoire of virulence factors, specifically adapted to its needs for invasion, colonization, replication, and survival in the host (**Table 1**). Survival of *P. aeruginosa* is supported by the secretion of toxins and proteases, including pyocyanin, exotoxin A, elastase, alkaline phosphatase, and phospholipase C (Lee et al., 2005; van’t Wout et al., 2015). Similar strategies are used by *B. cenocepacia* to invade and colonize host cells. Two zinc metalloproteases (ZmpA and ZmpB), phospholipase C, iron-chelating siderophores, and cable pili participate in this process (Sajjan et al., 1995; Darling et al., 1998; Chung et al., 2003; Corbett et al., 2003; Uehlinger et al., 2009). Besides the production of a range of extracellular enzymes (lipase, fibrinolysin, hyaluronidase, protease, elastase, etc.), little is known about virulence factors contributing to the pathogenesis of *S. maltophilia* (Bottone et al., 1986). The extracellular capsule, adhesion proteins (HMW1 and HMW2, opacity-associated protein A), pili, haemocin, and the IgA1 protease play a crucial role in the onset of the patient’s inflammatory response by *H. influenzae* (Rosadini, 2011; Kostyanev and Sechanova, 2012).

**Table 1:** Overview of the major virulence factors associated with the outer membrane or secreted by cystic fibrosis (CF) pathogens.

	*Pseudomonas aeruginosa*	*Burkholderia cenocepacia*	*Stenotrophomonas maltophilia*	*Haemophilus influenzae*
Proteases	LasB^2^, AprA^1^, AprX^1^, Staphylolysin LasA^2^, aminopeptidase PaAP^2^, protease IV^2^, LepA^5^, elastase^2^	ZmpA^2^, ZmpB^2^, MprA^2^	StmPr1^2^, StmPr2^2^, elastase^2^	IgA1 protease^5^
Lipases	LipA^2^, LipC^2^, phospholipase C^2^, PlcH^2^, PlcN^2^, ExoU^3^	Phospholipase C	Lipase	/
Toxins	Pyocyanin, exotoxin A^2^, Cif	Haemolysin	Zonula occludens toxin	/
Adhesion molecules	Chitin-binding protein CbpD^2^, pili, ExoS^3^, ExoT^3^, alginate, fimbriae, flagellin	Cable pili, flagellin, fimbriae	Flagellin, fimbriae	HMW1^5^, HMW2^5^, pili, Hap^5^, Hia^5^, Hsf^5^, opacity-associated protein A
Hydrolytic enzymes	Alkaline phosphatase, EstA^5^	Chitinase	Fibrinolysin, hyaluronidase, DNase, chitinase	Haemocin


*Virulence factors of *Achromobacter xylosoxidans* have not been characterized yet. Indices indicate a known association with a certain secretion system (number corresponds to the type of secretion system).*

## The Role of Bacterial Secretion Systems in CF Pathogenesis and Virulence

**FIGURE 1:**
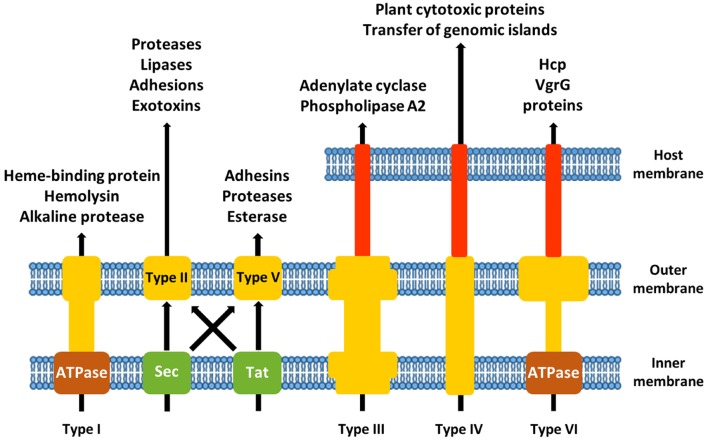
**Schematic overview of the different secretion systems of Gram-negative airway pathogens associated with cystic fibrosis (CF)**.

Bacterial virulence factors are delivered either in the extracellular environment or directly into host cells. Most Gram-negative CF pathogens possess one or more specialized secretion systems to accomplish this task. Eight different secretion systems have been identified (**Figure 1**). Type I [type I secretion system (T1SS)], type III [type III secretion system (T3SS)], type IV [type IV secretion system (T4SS)], and type VI [type VI secretion system (T6SS)] secretion pathways use a single energy-coupled step to transport proteins across both the inner and outer membranes. The outer membrane-spanning type V secretion system (T5SS) and the double membrane-spanning type II secretion system (T2SS) translocate substrates that first have been transported into the periplasm by the Sec or Tat machinery (Costa et al., 2015). Type VII secretion system (Type VII) is restricted to Gram-positive bacteria and will not be discussed here. The type VIII secretion system (type VIII) refers to the curli biogenesis pathway (Chapman et al., 2002).

### T1SS

The type I secretion machinery is composed of an inner membrane associated ATP-binding cassette protein (which recognizes the secretion signal of the substrate), a membrane fusion adapter protein and a TolC-like outer membrane protein (Wandersman, 1996). Substrate proteins are often very acidic and contain distinctive glycine-rich repeats that bind Ca^2+^ ions (Baumann et al., 1993). Most of the transported proteins also contain repeats with a high degree of homology to adhesion molecules, suggesting a role for T1SS substrates in adherence (Hinsa et al., 2003).

The heme-binding protein HasAp from *P. aeruginosa*, important for iron acquisition, is an example of a protein secreted by T1SS (Letoffe et al., 1998). A second T1SS in *P. aeruginosa* is responsible for the secretion of the alkaline proteases AprA and AprX (Guzzo et al., 1991; Duong et al., 2001). In *B. pseudomallei*, the major haemolysin is exported through a T1SS (Harland et al., 2007). Three T1SS clusters are present in the genome of *S. maltophilia* (Rocco, 2011), a potential substrate being the virulence-associated membrane protein Ax21 (Ferrer-Navarro et al., 2013).

### T2SS

The T2SS is important for the secretion of hydrolases. It consists of an outer membrane complex, a periplasmic pseudopilus, an inner membrane platform and a cytoplasmic ATPase. Substrates are transported into the periplasm as unfolded or folded proteins by the SecYEG translocon or the Tat transporter, respectively (Costa et al., 2015). Interaction of the T2SS with its substrates presumably occurs through recognition of a structural motif, rather than a linear secretion signal (Lu and Lory, 1996; Sauvonnet and Pugsley, 1996; Francetic and Pugsley, 2005).

In *P. aeruginosa*, the major extracellular protease LasB is secreted by the T2SS and is responsible for elastin degradation and cleavage of surfactant protein D, an important immune system protein (Olson and Ohman, 1992; Alcorn and Wright, 2004). Staphylolysin LasA, aminopeptidase PaAP, and protease IV are other examples of type II secreted proteinolytic enzymes in *P. aeruginosa* (Olson and Ohman, 1992; Engel et al., 1998; Cahan et al., 2001). Another important family of T2SS substrates in this pathogen are lipases, like LipA, LipC, phospholipase C, PlcH, and PlcN, which are targeting the host membrane (Diaz-Laviada et al., 1990; Ostroff et al., 1990). CbpD, a T2SS-dependent chitin-binding protein, could serve as an adhesin, mediating colonization of eukaryotic cells (Folders et al., 2000). The type II secreted exotoxin A is responsible for ADP-ribosylation of elongation factor 2, resulting in protein synthesis inhibition and cell death (Allured et al., 1986). Also the *B. cenocepacia* zinc-dependent metalloproteases, ZmpA and ZmpB, are T2SS substrates (Nakazawa, 1996). They cleave antimicrobial peptides involved in innate immunity, like β-defensin-1, cathelicidin LL-37, elafin, and secretory leukocyte inhibitor (Kooi and Sokol, 2009). *S. maltophilia* possesses two T2SS, Gsp and Xps (Karaba et al., 2013). The serine proteases StmPr1 and StmPr2 are substrates of the Xps T2SS and mediate degradation of extracellular matrix proteins (DuMont et al., 2015). *H. influenzae* does not contain the genes required to build a functional T2SS (Cianciotto, 2005).

### T3SS

Bacterial T3SS are nanomachines capable of injecting effector proteins into the cytoplasm or cell membrane of eukaryotic target cells, and are therefore also called injectisomes (Cornelis, 2006). The system consists of a double-membrane-spanning base composed of stacked rings and a needle-shaped filament that extends into the extracellular space (Marlovits et al., 2004). Different translocator proteins are first transported through the needle and inserted into the eukaryotic cell membrane to form a pore of about 2.8–3.0 nm (Dacheux et al., 2001; Schoehn et al., 2003). Effectors contain a non-cleavable N-terminal secretion signal and are targeted to the secretion machinery in an unfolded state (Cornelis, 2006).

Known T3SS effectors of *P. aeruginosa* include ExoS and ExoT, both containing a GTPase-activating function and an ADP-ribosyltransferase activity. By acting on the actin cytoskeleton, they are able to protect *P. aeruginosa* from phagocytosis (Barbieri and Sun, 2004). Accumulation of cyclic AMP in host cells is caused by the action of ExoY, an adenylate cyclase (Yahr et al., 1998). ExoU is responsible for acute cytotoxicity and lung tissue damage by its phospholipase A2 activity. Together with ExoS, it prevents interleukin production by alveolar macrophages and modulates the early inflammatory response (Sato and Frank, 2004). A T3SS mutant of *B. cenocepacia* was attenuated in virulence in a murine model of infection, which indicates a role for the T3SS in evasion of the host immune system (Tomich et al., 2003). Currently, no effectors have been identified for this species. T3SS genes are not present in *S. maltophilia* (Crossman et al., 2008) or *H. influenzae* (Harrison et al., 2005).

### T4SS

Like the T3SS, the T4SS is composed of a core complex spanning the inner and outer membrane and a pilus that protrudes into the extracellular environment (Christie et al., 2014). The secretion signals needed for translocation of effector proteins are generally localized at the C-terminus and consist of clusters of hydrophobic or positively charged residues (Alvarez-Martinez and Christie, 2009).

Two T4SSs with different functions are present in *B. cenocepacia*. The first is located on a 92 kb plasmid and is responsible for secretion of plant cytotoxic proteins. It also plays a role in the intracellular survival of *B. cenocepacia* in phagocytes. The second T4SS is chromosomally encoded and might be involved in plasmid mobilization, although the exact function is still unknown (Zhang et al., 2009). T4SS effectors of *Xanthomonas citri*, a close relative of *S. maltophilia*, have the capability of killing other bacterial species, thereby conferring a selective growth advantage in mixed bacterial communities (Souza et al., 2015). Whether the T4SS of *S. maltophilia* has a similar function, remains unknown. *H. influenzae* and *P. aeruginosa* do not contain a conventional T4SS.

A unique feature of the T4SS is that it can also transport nucleic acids. *P. aeruginosa* and *H. influenzae* possess one or more genomic island-associated T4SSs (GI-T4SS) that play a crucial role in horizontal gene transfer (HGT) of integrative and conjugative elements (ICEs; Juhas et al., 2007a). ICEs not only contain genes required for excision/integration and various accessory genes, but they often also harbor a T4SS, which completes the machinery for efficient transfer from donor to recipient cell (Juhas et al., 2008; Wozniak and Waldor, 2010; Guglielmini et al., 2011). A considerable part of the accessory genes are involved in antibiotic resistance or virulence. ICEHin1056 of *H. influenzae* carries ampicillin, tetracycline and chloramphenicol resistance genes (Juhas et al., 2007b), while PAPI-1 of *P. aeruginosa* encodes CupD type fimbriae essential for attachment and the PvrSR/RcsCB regulatory system involved in biofilm formation and antibiotic resistance (Mikkelsen et al., 2013). The chromosomally encoded T4SS of *B. cenocepacia* was also linked to plasmid mobilization (Zhang et al., 2009). Taken together, these mechanisms of HGT pose a major threat to our ability to combat infections occurring in CF patients by potentially transforming the lung microbiota into an antibiotic resistant community.

### T5SS

The T5SS is a single-membrane-spanning system that secretes virulence factors and mediates cell-to-cell adhesion and biofilm formation. The substrates are fused to their secretion pore to form a single polypeptide, also known as autotransporter. Unfolded autotransporters are delivered to the periplasm via the SecYEG translocon. The exoproteins either remain associated with the outer membrane or are released in the extracellular environment after proteolytic cleavage (Leo et al., 2012). In a second type of T5SS, two-partner secretion (TPS), the substrate or passenger domain and the pore-forming domain are two separate proteins.

There is only one known autotransporter in *P. aeruginosa*, i.e., EstA. It can hydrolyze glycerol esters through its esterase activity and is involved in the production of rhamnolipids, cell motility and biofilm formation (Wilhelm et al., 2007). Three TPS systems have been characterized in *P. aeruginosa*: the LepA/LepB system, in which LepA is a protease activating NF-κB through digestion of PAR receptors (Kida et al., 2008), the CupB system, involved in the assembly of CupB fimbriae (Ruer et al., 2008) and the PdtA/PdtB system, where PdtA is related to High Molecular Weight (MWH) adhesins (Faure et al., 2014). The genome of *B. cenocepacia* J2315 contains four T5SS, two of them contain pertactin domains involved in adhesion, and the other two contain haemagglutinin repeats (Holden et al., 2009). Haemagglutinin autotransporters are also present in *S. maltophilia* (Ryan et al., 2009). The HMW1 and HMW2 from *H. influenzae* are also TPS systems. The *H. influenzae* Hap, Hia, and Hsf autotransporters mediate bacterial aggregation and microcolony formation and promote adherence to epithelial cells and extracellular matrix proteins (Fink et al., 2003; Spahich and St Geme, 2011). Another T5SS substrate is the IgA protease, responsible for degradation of the major mucosal immunoglobulin (Fernaays, 2008).

### T6SS

The type VI secretion machinery consists of a membrane complex and a tail complex, composed of structural elements that are equivalent to contractile phage tails (Basler et al., 2012). Although the T6SS plays a major role in the pathogenesis toward eukaryotic cells, it can also be used to target other bacteria in polymicrobial infections (Ho et al., 2014). Three T6SS are present in *P. aeruginosa*, but only two major substrates have been identified so far, Hcp and VgrGs. Hcp is believed to form nanotubes on the bacterial surface, which may allow transport of other T6SS effectors (Ballister et al., 2008). VgrGs could form trimeric complexes puncturing membranes allowing the passage of other proteins (Leiman et al., 2009). The *B. cenocepacia* T6SS modulates actin cytoskeleton dynamics and NADPH oxidase complex assembly, also through the action of Hcp and VgrGs (Pukatzki et al., 2007). *S. maltophilia* and *H. influenzae* do not contain T6SS genes.

## Membrane Vesicles

Secretion of membrane vesicles (MVs) by both Gram-negative and Gram-positive bacteria is now considered as a true secretion system. The membranous nanoparticles are pinched off from the cell surface and carry membrane-associated and soluble proteins, nucleotides, and other molecules into the extracellular environment. MVs are involved in a series of biological functions, including nutrient acquisition, iron scavenging, antibiotic resistance and biofilm formation (Haurat et al., 2015).

Membrane vesicles contribute to pathogenesis by delivering virulence factors and/or through modulation of the host immune system (Schwechheimer and Kuehn, 2015). *P. aeruginosa* MVs enable long-distance delivery of multiple virulence factors including alkaline phosphatase, hemolytic phospholipase C and Cif, a toxin that inhibits CFTR-mediated chloride secretion in the airways (Bomberger et al., 2009). Cif also enhances ubiquitination and subsequent degradation of the transporter associated with antigen processing (TAP), reducing MHC class I activation (Bomberger et al., 2014). Secretion of MV-associated hydrolases like (metallo)proteases, (phospho)lipases and peptidoglycan-degrading enzymes was also shown in *B. cenocepacia* (Allan et al., 2003). *H. influenzae* MVs activate B-cells in a T-cell independent manner, possibly creating a diversion on the adaptive immune system and promoting survival within the host (Deknuydt et al., 2014).

Several studies highlighted the importance of MVs in antibiotic resistance. Exposure of *S. maltophilia* cells to β-lactam antibiotics led to a significant increase in MVs that are packed with β-lactamases (Devos et al., 2015). These MVs are capable of degrading β-lactams extracellularly, and even increase the β-lactam tolerance of the species *P. aeruginosa* and *B. cenocepacia* (Devos et al., 2016). Furthermore, β-lactamases were found in MVs of *P. aeruginosa* and *H. influenzae*, indicative for a general mechanism to respond to β-lactam stress (Ciofu et al., 2000; Schaar et al., 2011). MVs can also mediate export of antibiotics or extracellular capturing of antibiotics. When *P. aeruginosa* is treated with the aminoglycoside gentamycin, it secretes gentamycin-containing MVs. These MVs also contain peptidoglycan hydrolase and were shown to be bactericidal against *B. cenocepacia* (Allan and Beveridge, 2003). Finally, MVs can aid in the inter- and intraspecies spread of resistance genes (Schwechheimer and Kuehn, 2015).

## Secretion Systems As Targets For Anti-Infective Drugs

Development of novel therapies is crucial to manage the spread and impact of these pathogens on CF patients. Classical antibiotics mostly exert their function by inhibiting the growth of bacteria through interference with cell wall biogenesis, DNA replication, transcription, and protein synthesis (Baron and Coombes, 2007). Unfortunately, the rate at which resistance against these traditional antibiotics emerges is alarming, partly due to the rise of mutations in the genes coding for antibiotic targets. Secretion system inhibitors are a novel class of anti-infectives that do not inhibit bacterial growth *per se* and therefore do not provoke selection for mutations causing resistance. Another advantage is the fairly high degree of conservation of these systems between a whole range of Gram-negative pathogens. Since secreted effectors often play a major role in immune evasion, targeting these important bacterial virulence mechanisms may restore pathogen clearance by the host’s own immune system.

Kauppi et al. (2003) found that a family of acylated hydrazones of different salicylaldehydes can inhibit the T3SS at the level of substrate secretion/translocation. The related halogenated salicylaldehydes are capable of inhibiting the transcription of genes encoding T3SS components (Kenny et al., 1997). Thiazolidinones were found to target the formation or assembly of the T3SS needle apparatus. These compounds could also inhibit the T2SS in *Pseudomonas* and the type IV pili secretion system of *Francisella*, therefore it is hypothesized that they might act on the conserved outer membrane secretin (Felise et al., 2008; Kline et al., 2009). Other promising targets are the energy-generating ATPases of T2SS and T4SS (Sayer et al., 2014), the accessory lytic transglycosylases of T2SS, T3SS, and T4SS (Koraimann, 2003) and the translocated effector proteins (Coburn et al., 2007; Figueira et al., 2013; Kidwai et al., 2013). By inhibiting T4SS-dependent secretion, horizontal transfer of antibiotic resistance genes could be reduced.

## Concluding Remarks

With as many as 90% of CF patients dying of fatal lung infections every year, it is crucial to find means to eradicate or at least control the growth and spread of these major CF pathogens. Secretion systems provide a useful target, since their effector proteins are responsible for a wealth of host cell compromising actions. Due to the fairly high degree of conservation in the composition of these secretion systems, an inhibitor has the potential to target a whole array of Gram-negative pathogens. Because the growth of the pathogens is unaffected by such compounds, the risk for resistance development is highly reduced. It is therefore essential to keep investing in the identification of novel effector proteins and structural elements of secretion systems, as well as in ways to block secretion of virulence factors and MVs.

## Author Contributions

SoD wrote the chapters on secretion systems. SiD wrote the chapter on outer membrane vesicles. BD edited the manuscript and is the supervisor of the two other authors.

## Conflict of Interest Statement

The authors declare that the research was conducted in the absence of any commercial or financial relationships that could be construed as a potential conflict of interest.
